# Precision of the Wilson corrective osteotomy of the first metacarpal base using specific planning and instruments for treatment of basal thumb arthritis

**DOI:** 10.1007/s00402-022-04430-4

**Published:** 2022-04-09

**Authors:** Philipp Kriechling, Lisa Reissner, Christoph Zindel, Octavian Andronic, Andreas Schweizer

**Affiliations:** Department of Orthopaedics, Balgrist University Hospital Zurich, Forchstrasse 340, 8008 Zurich, Switzerland

**Keywords:** Arthritis, Basal joint, Metacarpal extension osteotomy, Thumb, Wilson osteotomy

## Abstract

**Introduction:**

Arthritis of the basal thumb is a relatively common condition also affecting younger patients. Wilson et al. described a 20°–30° closing wedge osteotomy of the first metacarpal bone to unload the trapeziometacarpal joint. It was the purpose of this study to analyze the clinical and radiographic outcome of patients who underwent proximal extension osteotomy of the first metacarpal bone using patient-specific planning and instruments (PSI).

**Methods:**

All patients who underwent proximal metacarpal osteotomy for basal thumb arthritis at our tertiary referral center were retrospectively included. The patients underwent preoperative planning using computed tomography and 3D segmentation to build patient-specific guides and instruments for the operative treatment. Stable fixation of the osteotomy was achieved by internal plating. The inclusion criterion was a minimum follow-up of 1 year with clinical examination, including the Michigan Hand Outcomes Questionnaire (MHQ), and computed tomography to validate the correction. Complications and reinterventions were recorded.

**Results:**

A total of eight Wilson osteotomies in six patients could be included at a mean follow-up duration of 33±16 months (range, 12 to 55 months). The patients were 49±8 years (range, 36 to 58 years) at the surgery and 88% were female. The postoperative MHQ for general hand function was 77±8 (range, 45 to 100) and the MHQ for satisfaction was 77±28 (range, 17 to 100). The working status was unchanged in 7/8 hands (6/7 patients). Radiographic analysis revealed successful correction in all cases with unchanged Eaton–Littler stage in 7/8 hands. No complications were recorded.

**Conclusion:**

The combined extending and ulnar adducting osteotomy using patient-specific guides and instrumentation provides an accurate treatment for early-stage thumb arthritis.

**Level of evidence:**

Type IV—retrospective, therapeutic study.

**Supplementary Information:**

The online version contains supplementary material available at 10.1007/s00402-022-04430-4.

## Introduction

Arthritis of the basal thumb is a relatively common condition also affecting younger patients [1]. A prevalence of up to 37% in people older than 80 years of age was described for basal thumb arthritis [2]. Despite that most patients can be treated conservatively at the early Eaton and Littler stages [3], higher degrees of osteoarthritis often need more elaborate treatment strategies like resection of the trapezium with or without ligamentous reconstruction and tendon interposition [2].

Wilson et al. described a 20°–30° closing wedge osteotomy of the first metacarpal bone to unload the trapeziometacarpal joint [4]. The authors reported satisfying clinical results for their patients [5]. Other studies also supported those first data, which have recently been extended to include long-term results [6–12].

Despite the promising outcome data reported, no study analyzed the achieved osteotomy using computed tomography. All existing studies have planned the osteotomy to be performed only indirectly based on the thickness of the wedge to be removed. Further, no studies using patient-specific planning and instruments (PSI) exist so far. We always performed a 20° extending and 5° ulnar adducting osteotomy according to a former 3D analysis of the first carpometacarpal joint, aiming to achieve an optimal joint surface contact in pinch grip position without disturbing ROM in Flexion.

Therefore, this study aimed to describe and analyze the accuracy of utilizing PSI to achieve the planned amount of correction and orientation of the closing wedge osteotomy and report clinical and radiographic outcome measures. We hypothesized that the planned osteotomy could be achieved using PSI with satisfying patient-reported outcomes measures following surgery.

## Material and methods

### Ethical approval

This study was approved by the cantonal ethics committee of the University of Zurich (Switzerland) (ID 2020-01888) and conducted following the Helsinki Declaration.

### Patients

All patients who underwent dorsal extension/ulnar adduction osteotomy of the first metacarpal bone for basal thumb arthritis between 2016 and 2018 at our tertiary referral hospital were retrospectively included in the analyses. The inclusion criteria were early-stage basilar osteoarthritis graded as Eaton and Littler [3] stage 1 or 2, a minimum follow-up of 1 year following surgery with complete clinical and radiographic follow-up (including computed tomography), signed informed consent, and patients with minimum age of 18 years. Exclusion criteria were previous surgical procedures at the thumb, pregnancy, depression and anxiety disorders.

### Preoperative planning and PSI guides design

The patients underwent computed tomography (Siemens Somotom Edge Plus, Germany) of the hand in 0.5 mm slice increments before surgery. All hand scans were three-dimensionally segmented using a standard segmentation software and algorithm (MIMICS version 23, Leuven, Belgium). Consecutively, a dedicated planning software (CASPA; Balgrist CARD AG, Zurich, Switzerland) was used to plan the osteotomy and create the PSI guides as previously described in the literature [13]. Those guides were designed to be utilized for the osteotomy and placing the holes for the plate, which was attached following osteotomy and extension (technique see below). The osteotomy was planned as a closing wedge osteotomy of 20° dorsal extension and 5° ulnar adduction at the first metacarpal bone as previously described by Wilson et al. [4].

### Surgical technique

**Fig. 1:**
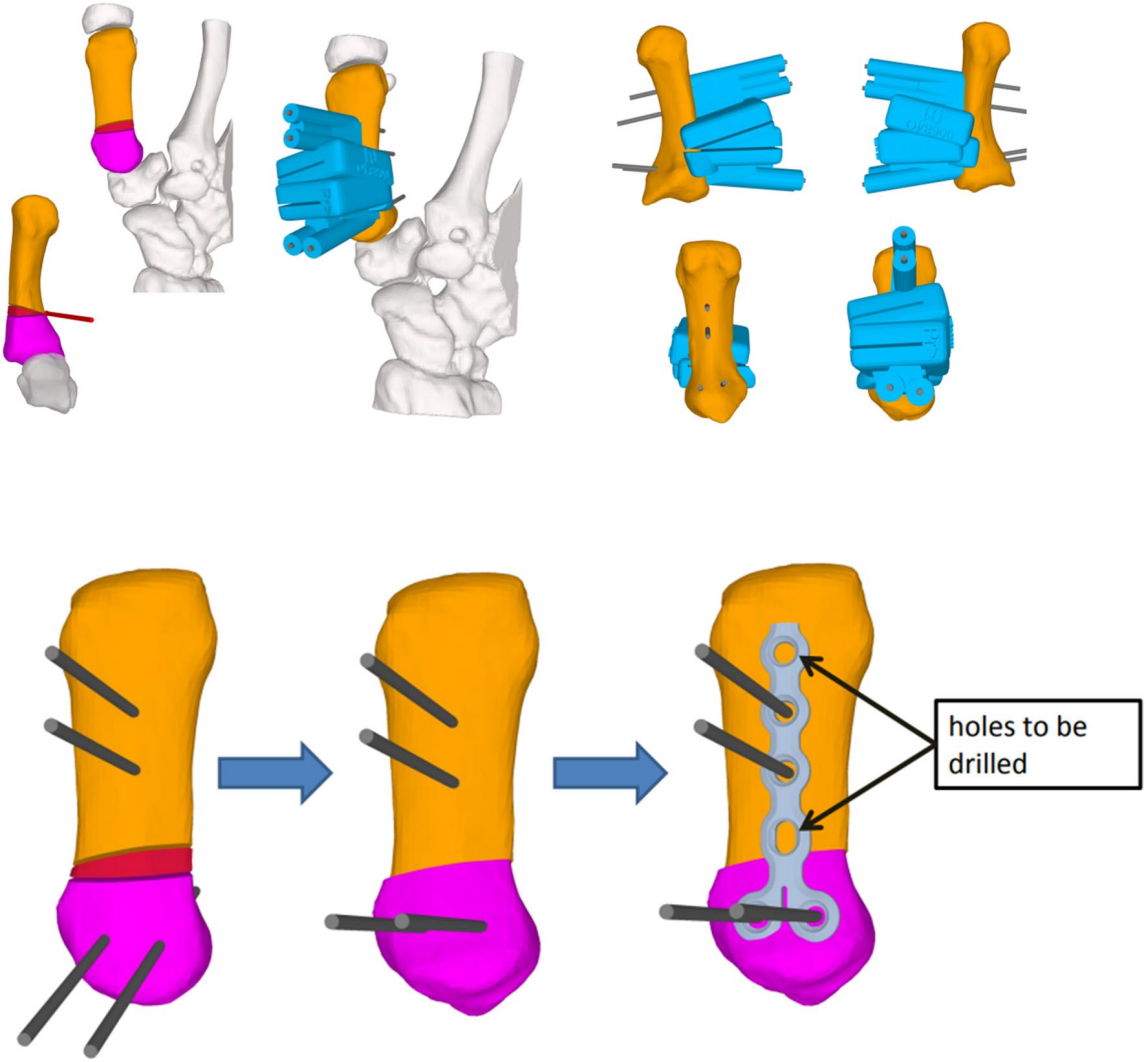
Surgical analysis and the plan using osteotomy guides. The osteotomy wedge (red) was planned as 20° dorsal extending and 5° ulnar adducting osteotomy. The patient-specific instrument guide (blue) was used to perform the planned osteotomy and to position the Kirschner wires. The holes of the Kirschner wires were later used to place the screws for plate fixation

All patients underwent surgery by a senior consultant in hand surgery in a standardized manner. The first metacarpal bone was approached dorsally to position the PSI osteotomy guides (Fig. 1). K-wires were inserted through predefined holes and the osteotomy was performed through the guide. Following the osteotomy, the guide was removed and a 1.5 LCP Compact Hand Condylar Steel Plate (DePuy Synthes, West Chester, Pennsylvania, USA) was attached through holes of the previously inserted k-wires. The vacant screw holes were drilled and locking screws were inserted. Subsequently, the K-wires were removed and the holes were filled with screws.

Postoperative care consisted of wearing a cast for 4–6 weeks before functional training. Return to sports was allowed from 8 weeks postsurgically.

### Clinical and radiographic analysis

The patients underwent a standardized clinical and radiographic evaluation at a minimum follow-up period of 1 year. One senior hand surgeon (L.R.) conducted a clinical assessment evaluating the overall outcome using the Michigan Hand Outcomes Questionnaire (MHQ) [14], the range of motion, key pinch strength (thumb vs. index finger) and three-finger pinch strength (thumb vs. index and middle finger) using a validated dynamometer (Pinch gauge, B&L Engineering, Santa Ana, USA). The patients were asked to rate the pain in rest and stress using a visual analog pain scale (VAS) from 0 to 10, with high values indicating more pain. Further, the patients were asked to rate the pain compared to the preoperative state as pain-free, improved, unchanged or worsened.

The MHQ is a self-reported questionnaire evaluating the six main categories of overall hand function, activities of daily living, pain, work performance, aesthetics, and patient satisfaction. Each item ranged from 0 to 100, with higher values indicating better results, except for pain. On the pain scale, higher values described more pain.

All patients received a second computed tomography (Siemens Somotom Edge Plus, Germany) postoperatively and three-dimensional (3D) models were segmented as described above. Consecutively, the segmented bone models were compared to preoperative models using a recently published 3D displacement description method [15]. This method described a 3D displacement of an obstacle with just two parameters: a pure shift and a pure rotation. For the preoperative to the postoperative analysis of the Wilson osteotomy, the method was applied to represent the deviation in height and rotation of the distal bone part of the osteotomy between the planning and the executed operation.

### Statistical analysis

Descriptive statistics are given as mean ± standard deviation (range, minimum to maximum). Preoperative and postoperative ordinal ranked data were compared using Wilcoxon rank sum testing. The significance level was set to *p* < 0.05.

## Results

### Patient sample

Eight osteotomies in six patients were performed. Postsurgery, the mean follow-up period was 33 ± 16 months (range, 12–55 months). The basic demographic data are presented in Table 1.

**Table 1:** Basic demographic data. Metric data are presented as mean ± standard deviation (minimum; maximum)

	Study group
Total number	8
Age at surgery (y)	49 ± 8 (36; 58)
Gender	1/8 male, 7/8 female
Dominant side	5/8 dominant, 3/8 not dominant
BMI (kg/m^2^)	25 ± 5 (20; 36)
Smoking	2 yes, 2 stopped, 4 never
Diabetes	1/8 (13%)
FUP	33 ±﻿ 16 (12; 55) months

B*MI* body mass index, *FUP* follow-up period, *kg* kilogram, *m* meter, *Y* years

### Clinical outcome analysis

All eight cases could be clinically analyzed postoperatively. The detailed data are displayed in Table 2 and supplemental Table 1. All eight osteotomies showed satisfying results comparing preoperative to postoperative pain scores in rest and stress. The pain in rest was described to be entirely resolved in four cases and improved in four. While stressing the thumb, the pain resolved completely in two cases and improved in six cases. The postoperative VAS pain levels were described as 0 ± 1 points (min, max; 0, 2 points) in rest and as 2 ± 3 points (min, max; 0, 7 points) in stress. All except one patient discontinued working in the same job as before surgery; the patient with a change in the work was a heavy manual labor worker.

**Table 2:** Postoperative outcome measures of the pathological and contralateral side. Metric data are presented as mean ± standard deviation (minimum; maximum)

	Postoperative pathological	Postoperative contralateral
Thumb flexion (°)	91 ± 4 (90; 100)	93 ± 5 (90; 100)
Thumb extension (°)	19 ± 6 (10; 25)	20 ± 14 (10; 40)
Thumb abduction (°)	41 ± 8 (30; 50)	38 ± 6 (30; 45)
Thumb adduction (°)	41 ± 6 (30; 50)	39 ± 9 (30; 50)
Opposition Kapandji	10 ± 1 (8; 10)	10 ± 1 (9; 10)
Retropulsion (°)	18 ± 6 (10; 25)	11 ± 5 (5; 15)
MCP flexion (°)	58 ± 7 (50; 65)	61 ± 9 (50; 70)
MCP extension (°)	9 ± 14 (− 20; 25)	5 ± 17 (− 10; 30)
Key pinch strength (kg)	6 ± 2 (3; 9)	8 ± 1 (3; 9)
Three-finger pinch strength (kg)	6 ± 2 (3; 8)	7 ± 2 (5; 9)
MHQ overall	77 ± 18 (45; 100)	90 ± 14 (70; 100)
MHQ ADL	84 ± 23 (40; 100)	98 ± 3 (95; 100)
MHQ work	71 ± 27 (25; 100)	
MHQ pain	24 ± 23 (0; 60)	9 ± 14 (0; 30)
VAS pain	2 ± 2 (0;6)	1 ± 1 (0; 3)
MHQ esthetic	87 ± 18 (50; 100)	83 ± 21 (56; 100)
MHQ satisfaction	77 ± 28 (17; 100)	91 ± 16 (67; 100)

Opposition was rated according to Kapandji et al. [24]; a score of 0 indicates no opposition of the thumb and a score of 10 indicates maximum opposition of the thumb. Key pinch strength measured the strength of the thumb against the index finger and the three-finger strength measured the strengths of the thumb versus the index and long finger. *ADL* activity of daily living, *kg* kilogram, *MCP* metacarpophalangeal joint, *MHQ* Michigan Hand Outcomes Questionnaire

### Radiographic outcome analysis

Radiographic outcome analysis is displayed in Table 3. The mean deviation from the planned to the performed osteotomy was 0.9 ± 0.7 mm in height and 4.2 ± 1.1° in combined rotation.

**Table 3:** Pre- and postoperative analysis for all eight hands showing the osteotomy height (mm) and the osteotomy angle (°)

	Surgical plan		Results		Deviation	
	Height (mm)	Angle (°)	Height (mm)	Angle (°)	Height (mm)	Angle (°)
1	3.86	20.00	3.24	17.21	0.62	2.79
2	4.05	20.02	4.6	14.15	0.55	5.87
3	5.38	20.00	3.42	14.29	1.96	5.71
4	3.62	20.01	4.42	15.89	0.8	4.12
5	3.37	20.00	5.47	15.94	2.1	4.06
6	4.65	19.99	3.79	17.45	0.86	2.54
7	4.79	20.01	4.81	24.54	0.02	4.53
8	2.72	20.00	3.16	16.42	0.44	3.58
Mean	4.06	20.00	4.11	16.99	0.92	4.15
SD	0.80	0.01	0.78	3.07	0.69	1.14

*Mm* millimeter, *SD* standard deviation

**Fig. 2:**
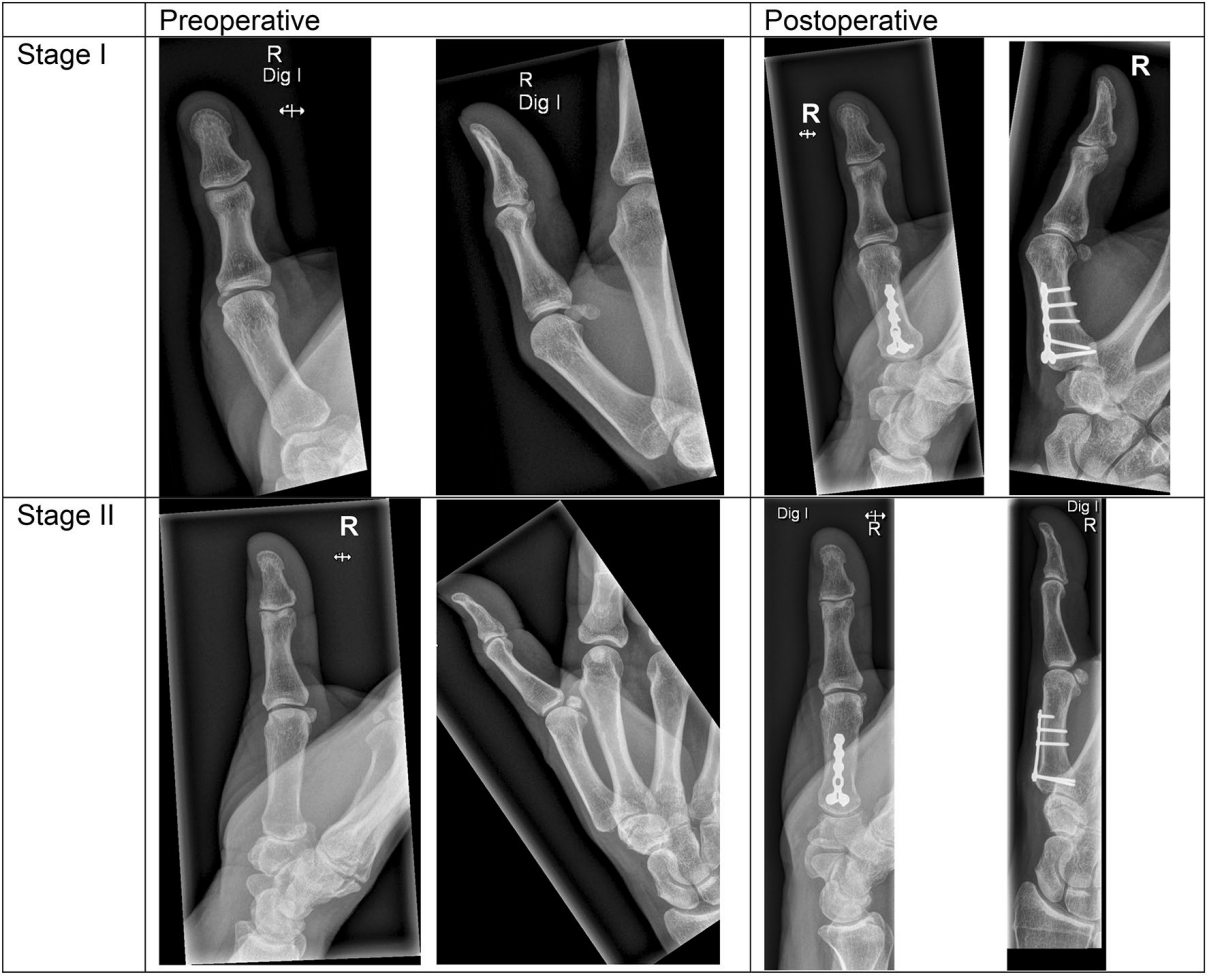
Shows thumb number 3 with basilar thumb arthritis assessed as Eaton and Littler stage I preoperatively **A** and postoperatively **B** and thumb number 7 with Eaton and Littler stage II preoperatively **C** and postoperatively **D**

The Eaton–Littler stage worsened in 1/8 of the cases from stage 1 to stage 2. Preoperatively, four patients were classified as stage I and 4 as stage II. Those changes were statistically not significant (*p* = 0.317). (Fig. 2, Supplemental Fig. S1).

### Complications and reinterventions

No complications were recorded. One patient (patient identification number 3 in the supplemental table) experienced a relapse of pain 10 months postoperatively without any traumatic event. He was further unable to continue his work as a heavy manual laborer. Nevertheless, a subjective pain reduction of 40% compared to preoperative could be achieved. Further injections to the joint could not improve the pain, neither could a resection of a symptomatic os sesamoideum. Interestingly, the patient was rated as Eaton-Littler-Stage I without progression.

Four reintervention surgeries were performed, of which three were the removal of the plate and one resection of symptomatic os sesamoideum in the patient with the lowest postoperative MHQ score.

## Discussion

The proximal closing wedge osteotomy for basal thumb arthritis was described by Wilson et al. [4] to unload the joint in the early stages of arthritis [16, 17]. A few studies, including some long-term data [6, 7, 11], reported satisfying clinical outcomes. However, all previous studies planned the osteotomy on preoperative plain X-rays and conducted an osteotomy according to the intraoperative measured osteotomy gap. No study analyzed the radiographic outcome using computed tomography.

We report the clinical and radiographic outcome of 8 extension osteotomies of the first metacarpal bone using PSI to treat basal thumb arthritis. The osteotomies were performed in 20° extension and 5° ulnar adduction to achieve an optimal joint surface contact in pinch grip position without disturbing the range of motion in flexion. The patients reported satisfying results with substantial improvement comparing the preoperative to postoperative state at a mean follow-up duration of 33 ± 16 months (range, 12 to 55 months). The mean postoperative overall MHQ was 77 ± 18 and the MHQ pain score 24 ± 23 or VAS 2 ± 2. MHQ patient satisfaction reached 77 ± 28. The radiographic analysis of the osteotomy using computed tomography and three-dimensional analysis revealed an average osteotomy angle of 17°, deviating 4.2° from the plan. The mean height of the osteotomy was 4.1 mm with a deviation of 0.9 mm from the plan.

We demonstrated the achievement of the planned osteotomy using PSI to carry out the desired osteotomy. No study in the current literature analyzed the outcome of the osteotomy using computed tomography. A previously published study [18] for corrective osteotomies of distal radial fractures revealed a residual error of 5.8 ± 3.6°, which is a bit more than in this study, possibly due to the higher amount of correction (32° vs. 20° planned correction). This is the first study to demonstrate results of a first metacarpal bone osteotomy using PSI. The advantages over conventional freehand techniques regarding surgical accuracy as well as long-term clinical outcome results have to be shown in future studies. PSI is known to have a high potential to achieve the planned results [19], which might become especially important in the area of the basilar thumb joint. [20] An exact planning of the correction with a successful shift in load stress could reliably improve patient outcomes compared to conventional techniques. Unfortunately, none of the previous studies describing the results of Wilson osteotomy conducted postoperative correction analyses. [5–9, 11, 12].

The clinical results of our study are comparable to the current literature, which is heterogeneous regarding surgical techniques and outcome analyses. The satisfaction mainly reached 70% to 90% according to the respective study [6, 7, 9–12]. Chou et al. [7] reported a satisfaction rate of 77% in 13 patients at a mean time of 10 years (range, 6 to 14). Gwynne et al. [8] published similar results on 28 hands with a follow-up duration of 34 months (range, 12–73), reaching a mean MHQ of 74 points postoperatively compared to 44 points preoperatively. This is comparable to our MHQ of 77 points at a similar follow-up period of 35 months in our patient cohort. Albeit the overall outcome is promising, it seems to depend on the duration of the follow-up. Tomaino et al. [12] described a satisfaction rate of 92% at 2.1 years (range, 0.5 to 3.8 years) in 12 patients and Parker et al. [11] described a satisfaction rate of 75% at 9 years (range, 6 to 13 years) in 8 patients.

The basal thumb joint's osteoarthritis stage progressed very slowly. According to the Eaton and Littler stage, only 1/8 (13%) went from stage 1 to stage 2. This fact may be explained by the short follow-up period of our cohort with 2.8 ± 1.3 years. Other authors described progression rates ranging from 38% at 9 years [11] and 10 years [7] to 83% at 12 years [6]. It has to be mentioned that the technique was described for patients with early-stage arthritis.

The complication rate in our cohort was very low. These results might be related to the surgical technique of internal fixation using a plate. Most authors [5, 7, 11, 12] used the original fixation method with an internal metal cable cerclage as described by Wilson et al. [4] Some authors [6, 8–10] used temporary or additional k-wire fixation. The latter method can possibly result in high infection rates as described by Bachoura et al. [6], who reported 28% with reoperation in 22%. Other described complications were dysesthesia, tendon rupture or insufficient correction. [6]

The corrective osteotomy of the first metacarpal bone has to be discussed in comparison to other surgical techniques, especially the trapezium resection arthroplasty with or without tendon interposition. This technique is described as standard procedure in late-stage basilar thumb arthritis (Eaton-Littler III/IV) with satisfying long-term results [21, 22]. Compared to the modified Wilson osteotomy, the resection arthroplasty is known to have a negative impact on pinch strength, which is essential for younger patients or laborers with manual work. [23] Currently, no optimal treatment algorithm is established regarding patients with early-stage arthritis (e.g., Eaton and Littler I/II) and exhausted conservative measures. Other techniques for early-stage basilar thumb arthritis include arthroscopy with debridement, denervation or autologous fat transfer; all techniques mainly provide symptomatic treatment for the patient [20]. In contrast, the modified Wilson osteotomy offers a curative approach in changing the joint articulation angle to unload the degenerated area of the joint surface. This study revealed a comparably low complication rate with high patient satisfaction levels in achieving the desired correction. The modified Wilson osteotomy might possibly be a therapeutic treatment for patients with early-stage basilar thumb arthritis. Finally, the surgical indication must be tailored to the patient's needs and daily demands.

Our study has several limitations. First, the study had a retrospective design with all the inherent biases. Secondly, no preoperative scores were available for comparison with the achieved results. Thirdly, we only reported on a small group of eight osteotomies with a minimum follow-up of 1 year. That might be explained by the new PSI technology, which we started using roughly 3 years ago for this relatively rare indication. Nevertheless, we decided to analyze these data with a minimum follow-up of 1 year to provide this knowledge and draw attention to the technique's strengths. Most of the studies in the current literature provide similar small patient numbers.

The proposed modification of the Wilson osteotomy using patient-specific guides and instrumentation provides a safe and successful treatment for early-stage arthritis of the thumb with low complication and reintervention rates. The combined dorsal extending and ulnar adducting osteotomy was performed with high accuracy in all patients and resulted in satisfying patient-reported outcome measures.

## Supplementary Information

Below is the link to the electronic supplementary material.

Supplementary file1. Table S1: Individual Michigan Hand Outcomes Questionnaire (MHQ) for each hand.

Supplementary file2. Figure S2: Presents the computer tomography of patient number 8 preoperatively and postoperatively.
