# Supplementary figures and images for: Precision of the Wilson corrective osteotomy of the first metacarpal base using specific planning and instruments for treatment of basal thumb arthritis
Source: Arch Orthop Trauma Surg. 2022 Apr 9;142(8):2103–10. doi: 10.1007/s00402-022-04430-4 (PMC9296388; doi:10.1007/s00402-022-04430-4)

Figure 2

| Preoperative | Postoperative |
| --- | --- |
| 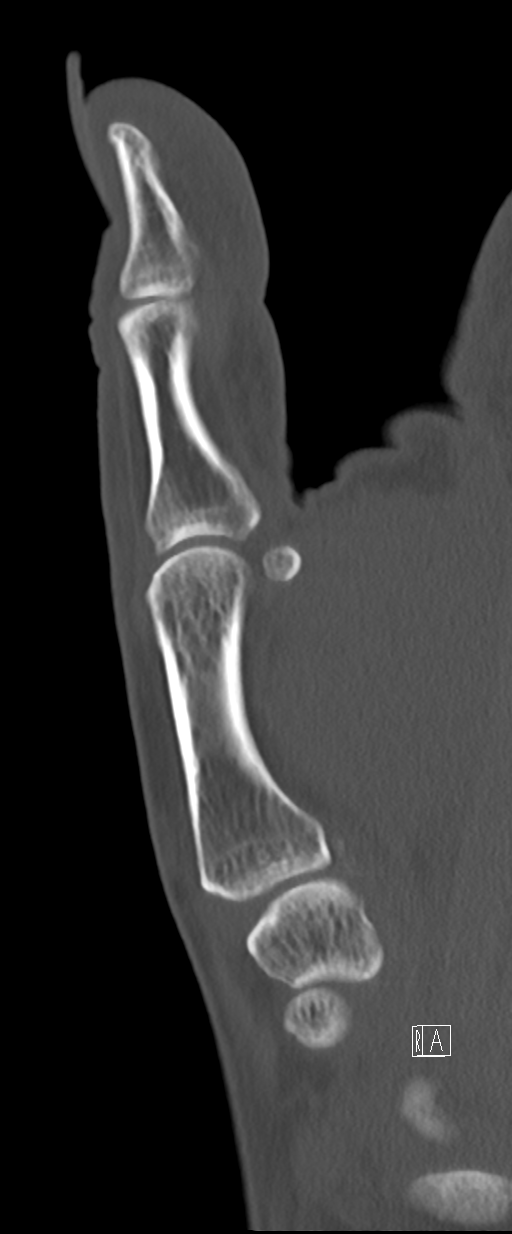 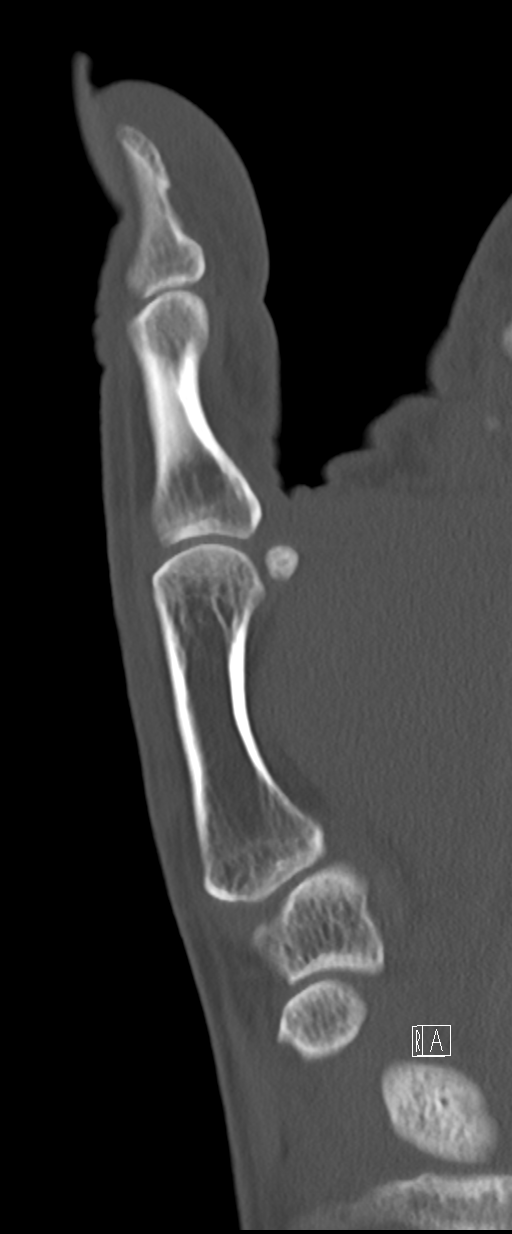 | 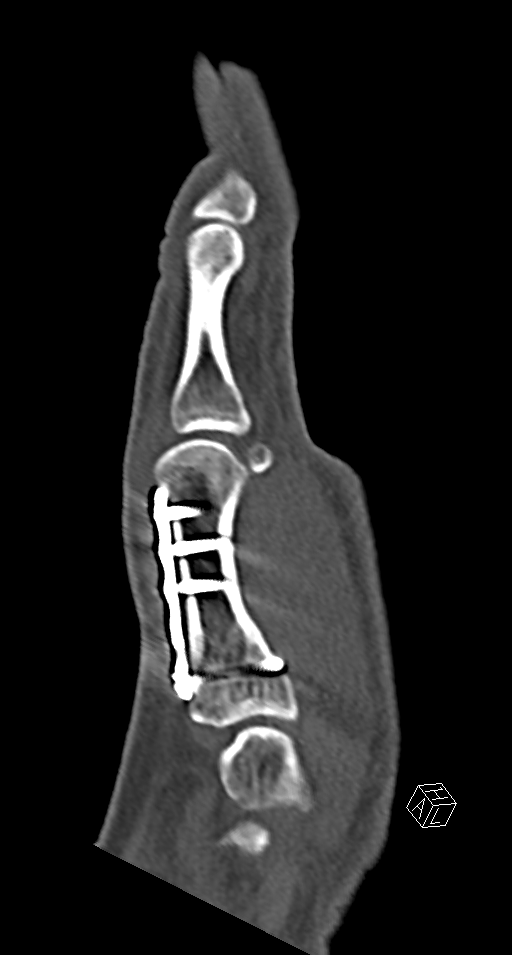 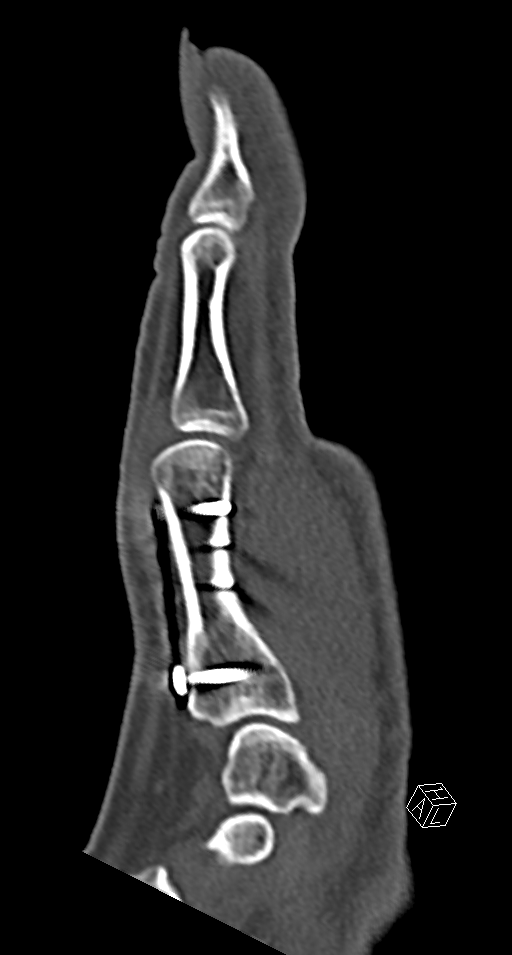 |
| 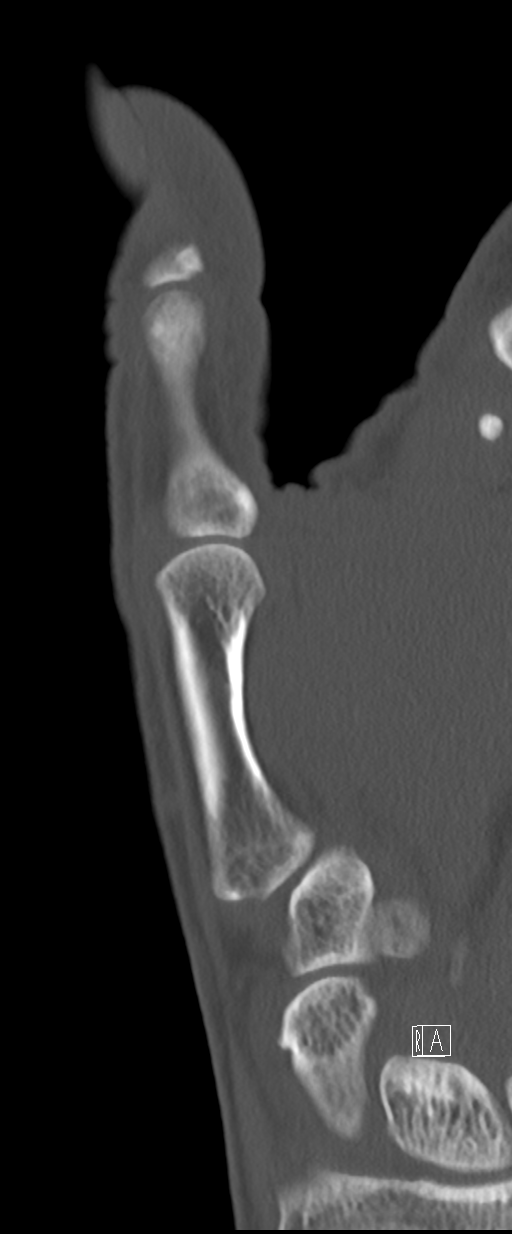 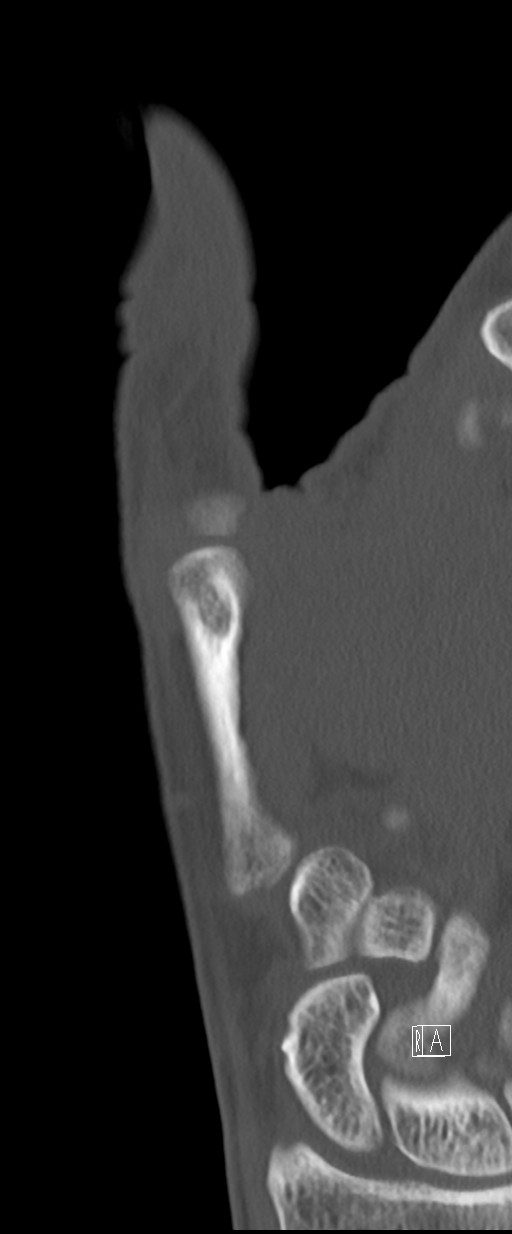 | 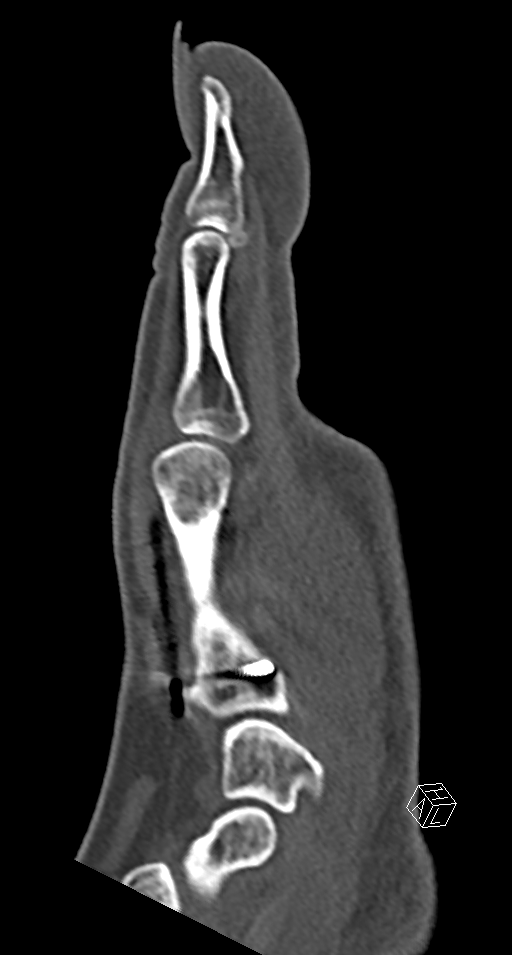 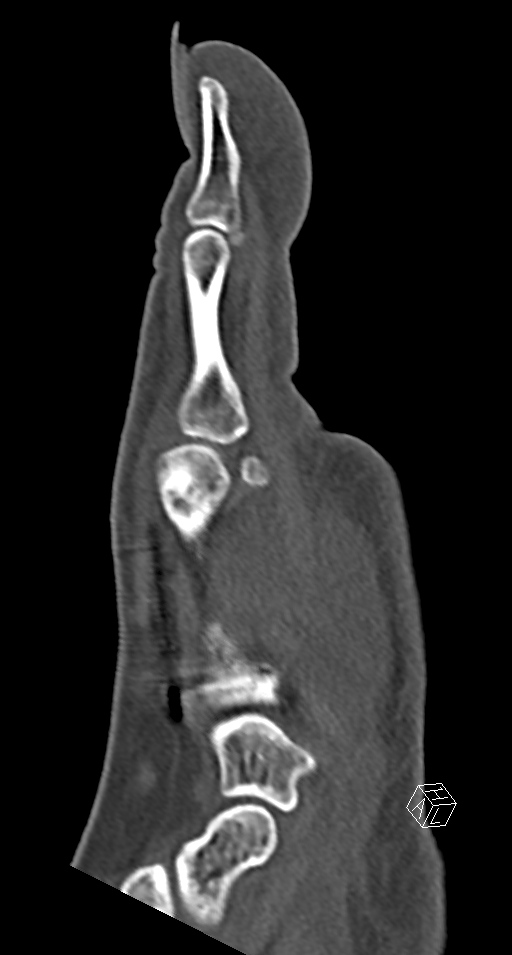 |

Supplement: Supplementary file 1 — Supplementary file1. Table S1: Individual Michigan Hand Outcomes Questionnaire (MHQ) for each hand. [file 402_2022_4430_MOESM1_ESM.docx]
