# Supplementary material for: Precision of the Wilson corrective osteotomy of the first metacarpal base using specific planning and instruments for treatment of basal thumb arthritis
Source: Arch Orthop Trauma Surg. 2022 Apr 9;142(8):2103–10. doi: 10.1007/s00402-022-04430-4 (PMC9296388; doi:10.1007/s00402-022-04430-4)
Supplement: Supplementary file 2 — Supplementary file2. Figure S2: Presents the computer tomography of patient number 8 preoperatively and postoperatively. [file 402_2022_4430_MOESM2_ESM.docx]

Supplemental Table 1

| **ID** | **Overall** | **ADL** | **Work** | **Pain** | **Aesthetic** | **Satisfaction** |
| --- | --- | --- | --- | --- | --- | --- |
| **1** | 80 | 95 | 100 | 10 | 100 | 100 |
| **2** | 100 | 100 | 100 | 5 | 93.75 | 100 |
| **3** | 45 | 40 | 25 | 55 | 50 | 17 |
| **4** | 70 | 55 | 60 | 60 | 100 | 79 |
| **5** | 70 | 95 | 60 | 25 | 100 | 63 |
| **6** | 75 | 95 | 60 | 10 | 75 | 75 |
| **7** | 75 | 95 | 60 | 25 | 75 | 75 |
| **8** | 100 | 100 | 100 | 0 | 100 | 100 |
|  |  |  |  |  |  |  |
| **Mean** | 77 | 84 | 71 | 24 | 87 | 76 |
| **Stdev** | 18 | 23 | 27 | 23 | 18 | 28 |
